# Perinatal Stressors and Consequences for Neonates with Critical Congenital Heart Disease

**DOI:** 10.3390/jcdd10120497

**Published:** 2023-12-15

**Authors:** Christina Ronai, Isabel Katlaps, Amanda Kim, Amy M. Valent, Kent L. Thornburg, Erin Madriago

**Affiliations:** 1 Department of Cardiology, Boston Children’s Hospital, Boston, MA 02115, USA; 2 Department of Pediatrics, Harvard Medical School, Boston, MA 02115, USA; 3 Department of Pediatrics, Oregon Health and Science University, Portland, OR 97239, USA; 4 Department of Obstetrics and Gynecology, Oregon Health and Science University, Portland, OR 97239, USA; 5 Center for Developmental Health, Knight Cardiovascular Institute, School of Medicine, Oregon Health & Science University, Portland, OR 97239, USA

**Keywords:** perinatal stress, congenital heart disease, fetal cardiology

## Abstract

Introduction: The prenatal diagnosis of congenital heart disease (CHD) is a traumatic event that can cause expectant parents to experience anxiety, depression, and toxic stress. Prenatal exposure to stress may impact neonatal postoperative outcomes. In addition, expectant parents may have other psychosocial stressors that may compound maternal stress. We investigated the relationship between stress in pregnancies complicated by prenatally diagnosed CHD and their neonatal outcomes. Methods: A pilot retrospective cohort study of pregnancies with prenatally diagnosed critical CHD (2019–2021) was performed. The collected data included pregnancy characteristics and neonatal and postoperative outcomes (including the need for exogenous corticosteroid treatment (ECT)). In order to quantify prenatal stressors, a composite prenatal stress score (PSS) was established and utilized. Results: In total, 41 maternal–fetal dyads were evaluated. Thirteen (32%) neonates had single-ventricle anatomy. The need for ECT after CHD surgery was associated with higher pregnant patient PSS (*p* = 0.01). PSS did not correlate with birthweight, infection, or hypoglycemia in the neonatal period. Conclusions: Prenatal stress is multifactorial; higher PSS is correlates with post-bypass ECT, suggesting that a stressful intrauterine environment may be associated with worse neonatal postoperative outcomes.

## 1. Introduction

Each year, 40,000 families receive the news that their fetus has congenital heart disease (CHD). Additionally, a quarter of these prenatally diagnosed congenital heart defects will be critical lesions requiring intervention in the first few days after birth. In fact, one in eighteen infant deaths are due to underlying CHD [1]. Therefore, it is not surprising that the diagnosis of CHD is a stressful event for parents and families. Besides the myriad of social stressors that expectant parents may be simultaneously contending with, including food insecurity, unemployment, discrimination, distance from medical care, and/or economic uncertainty, just undergoing a fetal echocardiogram has been shown to cause tremendous additive stress and anxiety [2]. That stress is only increased when the imaging confirms an abnormality [3,4]. Diagnosis of a fetal congenital heart defect leads to anxiety, depression, and traumatic stress in up to 65% of expectant parents given such a diagnosis [5,6,7]. Moreover, recent studies have demonstrated that the more severe the fetal anomaly is, the higher the perceived stress is in the pregnant patient [8].

Increasing evidence points to the prenatal developmental environment as a critical and unique time period in which a pregnant patient’s exposure to toxic influences leads to fetal adaptation and developmental programming of later chronic diseases in the offspring during adulthood [9,10,11,12,13,14]. A developmental origin of health and disease approach speculates that exposure to prenatal adversity and stress, hormonal influences, inflammatory processes, and other toxic exposures, including malnutrition, influence the pregnant patient–fetal environment and effectively, and often maladaptively, program the developing fetus. Placental dysregulation and epigenetic alterations can ultimately lead to growth restriction and preterm birth [15,16]. Gestational hypertension, pre-eclampsia, gestational diabetes, and smoking have been shown to be associated with increased postoperative mortality for infants with hypoplastic left heart syndrome [12]. Multiple studies have shown that chronic toxic stress in pregnant patients is associated with higher overall cortisol levels in pregnancy [17,18,19,20,21,22], which in turn results in disadvantageous fetal programming and adverse outcomes such as fetal growth restriction, preterm birth [16], and long-term health consequences in the infant [23]. Prenatal stress associated with lower socioeconomic status has been associated with changes in fetal brain development [5,24,25], and the effect of fetal programming on later vulnerability to cardiovascular and related diseases has also been well established [13].

For patients with CHD specifically, determining whether high amounts of prenatal maternal stress exposure (both from psychosocial stressors and diagnosis of fetal CHD) is predictive of post-surgical instability could aid in the development of a predictive tool to use for post-operative neonatal management. Therefore, we collected pilot data to investigate if there was an association between prenatal psychosocial stress and postnatal outcomes for infants who received a prenatal diagnosis of CHD. We hypothesized that dyads with higher prenatal stress would be more likely to require increased postoperative exogenous corticosteroid treatment (ECT).

## 2. Methods

We retrospectively collected pilot data in a cohort of all pregnancies complicated by a fetal diagnosis of critical CHD with live births that went on to require early cardiac intervention (within 30 days of birth) between 1 May 2019 and 1 May 2021 at Oregon Health & Science University. Critical CHD diagnoses included D-transposition of the great arteries, single ventricle (hypoplastic left heart syndrome, tricuspid atresia, double outlet right ventricle), total anomalous pulmonary venous return, truncus arteriosus, critical pulmonary stenosis, critical aortic stenosis, interrupted aortic arch, and aortic coarctation. We subdivided patients with coarctation into those who required median sternotomy versus lateral thoracotomy surgical approach. Patients were only excluded from our study if they were transferred to a different institution for delivery and postnatal management.

For those meeting inclusion criteria, we reviewed the pregnant patient’s electronic medical record (EMR) for sociodemographic information, perinatal and medical history, and social worker assessments. The data included obstetric history, number of current living children, domestic partner status, insurance status and type, race, ethnicity, preferred language, medical comorbidities, the presence or absence of smoking, the presence or absence of pre-eclampsia and gestational hypertension, the presence or absence of pregestational or gestational diabetes, gestational age at fetal diagnosis, and whether diagnosis occurred before or during the COVID-19 pandemic. The EMR for the infant with critical CHD was also reviewed for gestational age at birth, birthweight, genetic testing results, cardiac diagnosis, age at first intervention, surgery metrics (cardiopulmonary bypass vs. non-bypass requirements, bypass and cross-clamp time), catheter-based intervention, and postsurgical variables. The primary outcome evaluated was the need for ECT. Secondary outcomes included the presence/absence of neonatal hypoglycemia, the presence/absence of neonatal infection (determined via blood, urine, and cerebrospinal fluid culture results), the postoperative vasoactive inotropic score (VIS), the need for extracorporeal membrane oxygenation (ECMO), and in-hospital mortality.

To evaluate prenatal stress, we retrospectively examined the EMR of the pregnant patient looking specifically at obstetric and sub-specialist medical visits and prenatal social work assessment notes for seven psychosocial components routinely documented for clinical purposes. These components included 1. prenatal mental health diagnosis (e.g., depression, bipolar disorder, schizophrenia); 2. housing insecurity (current eviction notice due to lack of payment, cohabitation with friends/family due to lack of resources, current habitation in shelter) and/or food insecurity (receiving SNAP benefits or positive screen for food insecurity); 3. low income (either subjectively perceived or below 138% of the federal poverty level as defined by Medicaid eligibility); 4. the lack of sufficient social support including current or future child care; 5. legal involvement for pregnant subject or spouse (e.g., incarceration or current investigation); 6. unreliable transportation (e.g., no reliable vehicle and no public transportation); and 7. other potential stressors or barriers to recommended medical care (e.g., another child with medical complexity, limited English proficiency, or sources for discrimination). A single licensed clinical social worker then assigned each of these components a severity score from 0 (no concerns) to 3 (significant concerns). Finally, we created a cumulative prenatal stress score (PSS) by adding the individual component scores together. We defined the cumulative PSS as low if the cumulative score was 3 or less and high if greater than or equal to 4. Substance use (including tobacco, alcohol, and others) was considered separately and not part of the PSS.

Statistical analysis was performed with GraphPad Prism 9.1.2. Our primary outcome of interest was the use of ECT after intervention (surgical or catheter-based). Two-tailed *t*-tests were used to compare continuous variables, and chi-square tests were used to compare categorical variables. The study was approved by our institutional review board.

## 3. Results

In total, 41 pregnant subjects (17 to 43 years, mean 33 years) and their neonates met our inclusion criteria. There were no intrauterine fetal demises. Pregnant subject demographics are shown in Table 1. Subject race and ethnicity data are consistent with Oregon state population demographics. There were 20 subjects seen during the pre-COVID-19 era and 21 during the COVID-19 era.

Neonatal demographics are depicted in Table 2. The median gestational age at birth for the 41 infants was 38 1/7 weeks. Of the 41 infants, 17 (42%) were female, and 13 (32%) had single-ventricle anatomy (Table 2). Almost two-thirds (63.5%) of the infants required neonatal CHD surgery, and the remaining 15 (36.5%) required a neonatal catheterization procedure. There was no statistical difference between neonates that were in either the high- or low- PSS groups.

**Figure 1:**
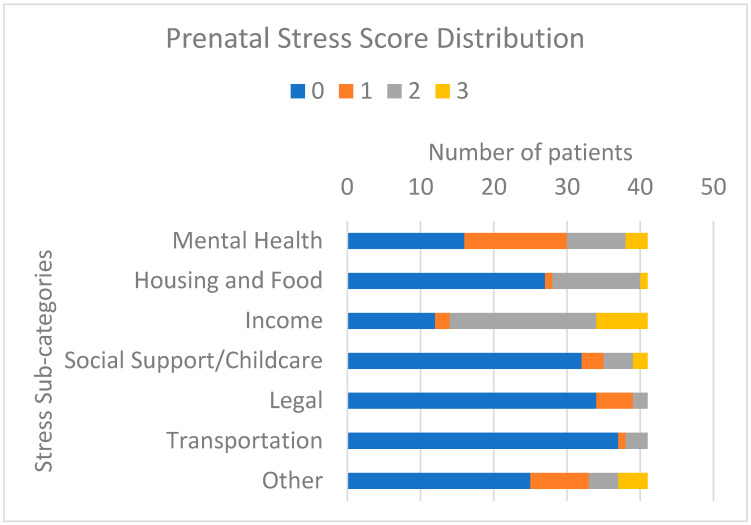
Prenatal stress score distribution by category among pregnancies complicated by fetal critical congenital heart disease.

The median cumulative PSS was 4, with subjects ranging from 0 to 12. Overall, 71% of subjects had concerns about income, 61% had additional mental health comorbidities, and 34% had concerns about housing/food (Figure 1). In addition, 27% endorsed substance use during pregnancy. Notably, subjects with private insurance had significantly lower PSS than those with public insurance (*p* = 0.0004).

For pregnant subjects with low or high cumulative PSS, there were no differences in gestational age at birth, birthweight, or number of neonates with single-ventricle anatomy (Table 3). Pregnant subjects’ individual stress sub-categories, the severity of prenatal CHD diagnosis (single ventricle vs. biventricular physiology), and counseling during the COVID-19 era did not correlate with postoperative outcomes. Additionally, the presence of gestational hypertension, pre-eclampsia, gestational diabetes, and maternal age did not correlate with the need for postoperative ECT.

**Figure 2:**
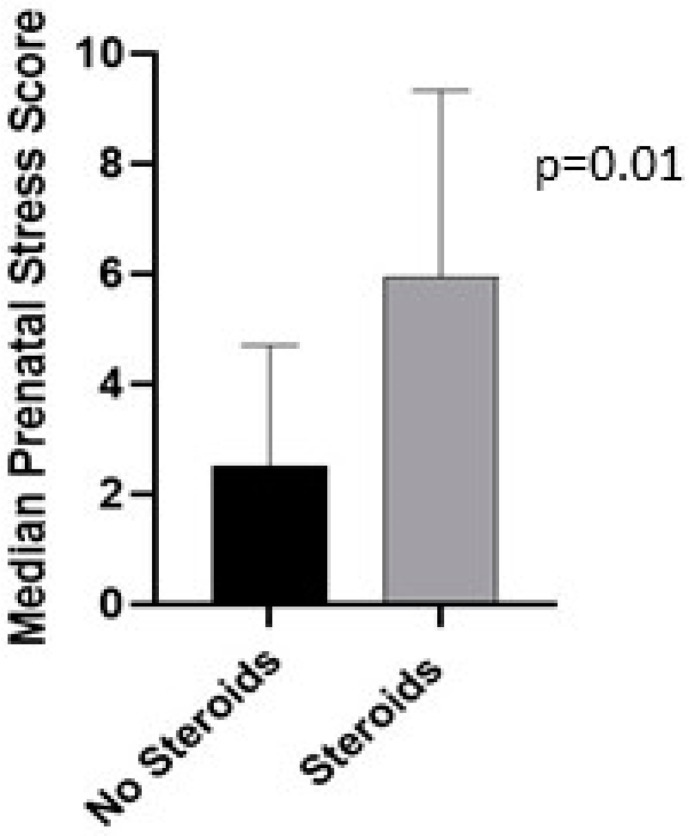
Median prenatal stress score comparison between neonates who did and did not require steroids after intervention.

There was a statistically significant difference (*p* = 0.01) between those with low or high PSS and postoperative need for ECT (Figure 2). Those who had higher prenatal stress scores were more likely to have neonates who required corticosteroids postoperatively for support. In comparison to the surgical group, none of the neonatal patients who underwent a cardiac catheterization procedure required steroid supplementation (*p* < 0.0001). Finally, surgical patients needing cardiopulmonary bypass were more likely to require postoperative steroids than those not requiring cardiopulmonary bypass (*p* 0.005). Documented reasons for ECT postoperatively were hypotension not responsive to increased vasoactives, low serum cortisol, and decreased ventricular function (Table 4). For this small cohort, there was no correlation between PSS and other neonatal or postoperative outcomes.

## 4. Discussion

Our pilot study demonstrates that prenatal stress is multifaceted and that higher PSS is correlated with a need for ECT after cardiac surgery. As knowledge increases about how the prenatal environment dynamically affects and potentiates the long-term determinants of health and disease, it is essential that we investigate the influences upon that environment. Stress, a ubiquitous factor in many pregnancies, is often accentuated in pregnancies with fetal anomalies and may have a pronounced effect on long-term outcomes for those children. Although prenatal detection and counseling have improved delivery planning [26] and surgical outcomes for infants with critical cardiac lesions, for some families, it comes with the cost of sustained parental psychological suffering [27]. While mitigating that distress has become a focus for the field in improving the quality of life for pregnant patients and families, the medical literature [27] suggests that stress leads to far greater and potentially longer-standing biological effects than we have previously accounted for in neonates as well.

For many years, the field of neonatal cardiac surgery has struggled with how best to predict which neonates will have insufficient cortisol response to the physiologic stress of surgery, resulting in a prospective randomized controlled trial evaluating the role of corticosteroids after neonatal heart surgery [28]. In pregnancies that are not associated with chronic stress and elevated cortisol levels, intermittent excess maternal cortisol is inactivated by placental 11β-HSD2 and converted to the metabolically inactive form, cortisone [29], thus preventing fetal exposure to high levels. However, when cortisol levels remain high over weeks or longer because of pregnant patient stress, the enzyme 11β-HSD2 is overwhelmed, and cortisol passes to the fetus where it influences development. The effect of high cortisol on the fetal hypothalamic pituitary adrenal axis (HPA) has not been well investigated but is postulated to be suppressed and therefore limited in its ability to generate endogenous cortisol. Therefore, some infants exposed to high cortisol in utero may have low cortisol levels for a period of time after birth. While this may not affect the vast majority of infants, those who are also suffering from CHD may be significantly affected.

Our pilot study argues for also looking backward toward the intrauterine environment as a potential marker of who may struggle with responding to the physiologic stress of a major surgery. Interestingly, pregnancy characteristics typically associated with an adverse uterine environment (gestational hypertension, pre-eclampsia, gestational diabetes, and smoking) were not associated with the need for postoperative ECT in our cohort. Instead, pregnant patients with higher overall composite stress scores were more likely to have infants that required corticosteroids after CHD surgery, suggesting a relationship between prenatal psychological stress and a resulting dysfunctional neonatal stress response system.

Cardiac surgery requiring cardiopulmonary bypass provokes a significant inflammatory response in neonates that necessitates an adequate adrenergic response. In our pilot cohort, those with catheter-based interventions (including those performed on patients with high-risk single-ventricle anatomy) did not require exogenous corticosteroid supplementation, and those not requiring bypass for surgical correction were less likely to need them than those undergoing cardiopulmonary bypass. Catheterization and non-bypass cases, while invasive, may not reach the threshold to overcome the intrinsic neonatal baseline stress response.

While we would expect significant dysregulation of the stress response to manifest in additional clinical needs, in this study, composite PSS did not correlate with other postnatal and postoperative outcomes such as neonatal hypoglycemia, neonatal sepsis, need for ECMO, or death. However, two of the three neonates requiring postoperative ECMO and all three of the neonates who died in our cohort were born to mothers who fell within the high-PSS group. Given that stress is additive, and stress originating from multiple sources (including pregnant patient complications such as pre-eclampsia and/or anxiety surrounding social stressors) is more likely to lead to physical and metabolic dysregulation [30], reassessing prenatal stress as a sum of its parts may be essential to further investigation.

There are several limitations to our pilot study. Our study population was limited to 41 pregnant patient–fetal dyads who were predominantly white, had access to a full spectrum of pregnancy options, were insured, and lived in the Pacific Northwest. Therefore, they may not be representative of society overall or individual reactions to different stressful situations. In addition, the cumulative PSS was meant to be all-inclusive but was not specifically validated and was determined retrospectively from prospectively administered psychological assessments. Moreover, we did not see a direct relationship between individual stress categories and the need for ECT. The stress categories were also equally weighted and may not universally reflect mutually exclusive stressors or may not reflect the true effect on individuals, and we were unable to perform multivariate analysis given our small sample size. Finally, we did not perform a quantitative assessment of cortisol levels in either the pregnant patient or the fetus. These pilot data demonstrate a need for prospective studies of larger cohorts utilizing both comprehensive qualitative and quantitative evaluations of the effect of stress on both the pregnant patient and subsequent infant outcomes.

In conclusion, this pilot study is a first step toward investigating whether stress in pregnancy is associated with adverse neonatal outcomes from surgery for critical congenital heart disease. Many patients reported socioeconomic concerns, including low income, mental illness, housing, and food insecurity, in addition to the stress of receiving the news about a critical heart lesion in their fetus. As those stressors added up, higher composite stress scores were correlated with post-bypass corticosteroid requirements for their infants, suggesting that a stressful intrauterine environment may be associated with an impaired neonatal response to physiologic stress. These data highlight the fact that prenatal stress is multifactorial, cumulative, and pervasive in this population and may lead to long-term detrimental effects not previously recognized.

## Figures and Tables

**Table 1:** Data on maternal demographic and perinatal characteristics.

Maternal Demographic and Perinatal Characteristics (n = 41)	
Age (years)	33 (17–43)
White race	32 (78%) *
Hispanic ethnicity	8 (20%) *
Interpreter needed	2 (5%)
BMI	30.6 (17.2–48.7)
Married/Domestic partner	36 (88%)
Number of other children (median and range)	2 (1–9)
Private insurance	19 (46%)
Uninsured	0
Tobacco use	9 (22%)
CHD Diagnosis during the COVID-19 era	21 (51%)
Gestational age at CHD diagnosis (weeks/days)	23 w 2 d (18 w 1 d–34 w 4 d)
Intrauterine demise	0
Gestational diabetes mellitus	12 (29%)
Hypertensive spectrum disorder (gestational hypertension, pre-eclampsia)	18 (44%)
Cesarean delivery	15 (37%)
Cumulative PSS Score	4 (0–12)

Data are expressed as median (range) or as a number (percentage). PSS (prenatal stress score), BMI (body mass index); * consistent with Oregon population demographics.

**Table 2:** Neonatal demographics.

Neonatal Demographics (n = 41)	Total Group	Low PSS (0–3) n = 18	High PSS (≥4) n = 23
Gestational age at CHD diagnosis (weeks/days)	23 w 2 d (18 w 1 d–34 w 4 d)	22 w 2 d (18 w 1 d–33 w 5 d)	24 w 2 d (20 w 0 d–34 w 4 d)
Gestational age at birth (weeks/days)	38 w 1 d (31 w 2 d–39 w 5 d)	37 w 6 d	38 w 5 d
Birthweight (kg)	3.1 (1.5–4.63)	3.2	3.1
Female	17 (41.5%)	8 (44%)	9 (39.1%)
Genetic abnormality	17 (41.5%)	7 (38.9%)	10 (43.5%)
Single-ventricle anatomy	13 (31.7%)	7 (38.9%)	6 (46.2%)
Age at intervention (days)	8 (2–54)	8 (2–48)	8 (5–54)
**Neonatal Procedures (n = 41)**	**Total**	**Low PSS**	**High PSS**
*Bypass* Arterial switch Stage 1 Norwood TAPVR repair Truncus arteriosus repair Interrupted aortic arch repair Aortic coarctation repair (median sternotomy) *Non-Bypass* Aortic coarctation repair (lateral thoracotomy)	26 (63.5%) 11 6 1 1 1 2 4	11 3 4 0 0 0 1 3	15 8 2 1 1 1 1 1
Bypass/Cross-Clamp Time (minutes)	189 (53–356) /109 (11–271)	148/77	188/124
*Catheterization procedures* Right ventricular outflow tract stent Ductal stent Balloon valvuloplasty No intervention	15 (36.5%) 3 9 2 1	7 1 4 1 1	8 2 5 1 0

Data are expressed as median (range) or as a number (percentage). PSS (prenatal stress score).

**Table 3:** Neonatal perioperative outcomes.

Neonatal Perioperative Outcomes	Total Group n = 41	Low PSS n = 18	High PSS n = 23	*p*-Value
Hypoglycemia	9 (22%)	4	5	NS
Pre-procedure infection concern	15 (36.6%)	7	8	NS
Post-procedure infection concern	15 (36.6%)	7	8	NS
Median initial vasoactive-inotropic score (26 patients)	9.5 (0–28)	9.5 (0–20)	10 (0–28)	NS
Steroid supplementation (surgery)	18 (43.9%)	5 (27.8%)	13 (56.5%)	*p* < 0.01
Steroid supplementation (catheterization)	0	0	0	NS
Mortality	3 (7.3%)	0	3	NS
ECMO	3 (7.3%)	1	2	NS

Data are expressed as median (range) or as a number (percentage), NS (not significant).

**Table 4:** Reason for steroid treatment.

Reason for Steroid Treatment	Total (18)	Low PSS (5)	High PSS (13)
Decreased heart function	3	0	3
Hypotension	4	1	3
Abnormal heart rhythm	2	0	2
Infection	0	0	0
Low serum cortisol	0	0	0
Reason not defined	9	4	5

## Data Availability

All data are presented in the main manuscript.
